# Cisplatin-loaded hollow gold nanoparticles for laser-triggered release

**DOI:** 10.1186/s12645-018-0041-9

**Published:** 2018-08-03

**Authors:** Chiyi Xiong, Wei Lu, Min Zhou, Xiaoxia Wen, Chun Li

**Affiliations:** 1 Department of Cancer Systems Imaging, The University of Texas MD Anderson Cancer Center, Houston, USA; 2 Present Address: School of Pharmacy, Fudan University, 826 Zhangheng Rd, Shanghai, 201203 China; 3 Present Address: Institute of Translational Medicine, School of Medicine, Zhejiang University, Hangzhou, 310029 China

**Keywords:** Cisplatin, Hollow gold nanoparticles, Near-infrared light, Controlled release, Photothermal conversion

## Abstract

**Background:**

Hollow gold nanoparticles (HGNPs) exposed to near-infrared (NIR) light yield photothermal effects that can trigger a variety of biological effects for potential biomedical applications. However, the mechanism of laser-triggered drug release has not been studied before.

**Methods:**

A tripeptide Ac-Glu-Glu-Cys-NH_2_ (Ac-EEC) was directly linked to the surface of HGNPs. The EEC-HGNPs conjugate was then complexed with cisplatin Pt(II) to give Ac-EEC(Pt)-HGNPs. Folic acid was introduced to the gold surface of Ac-EEC-HGNPs through a thioctic acid-terminated polyethylene glycol linker (F-PEG-TA) followed by complexation with Pt(II) to give F-Ac-EEC(Pt)-HGNPs. Laser treatment was instituted with a 15-ns pulsed laser at a repetition rate of 10 Hz. The released Pt(II) was quantified by inductively coupled plasma mass spectroscopy, and the nature of the released Pt-containing species was characterized by liquid chromatography–mass spectroscopy. The cytotoxicity was studied using the MTT assay.

**Results:**

Pt(II) was released from Ac-EEC(Pt)-HGNPs via two modes: (1) sustained release through an inverse ligand exchange reaction with chloride ions and (2) rapid release through cleavage of the Au–S bond between the tripeptide linker and Au surface upon NIR laser irradiation. The folate (F) conjugate of the nanoconstruct, F-Ac-EEC(Pt)-HGNPs, in combination with laser treatment showed a significantly greater effect on cell mortality against folate-overexpressing human epidermoid carcinoma KB cells than F-Ac-ECC(Pt)-HGNPs alone after 24 h of incubation.

**Conclusions:**

These results demonstrate that the photothermal property of HGNPs can be used for dual-modality photothermal therapy and NIR laser-triggered platinum-based chemotherapy.

## Background

Among the inorganic nanoparticles, gold nanoparticles have garnered the greatest attention for biomedical applications. Gold nanoparticles exhibit a unique and tunable optical property, termed surface plasmon resonance (SPR), which accounts for their photothermal effects (Jain et al. 2007). Gold nanoparticles also exhibit excellent biocompatibility (Bhattacharya and Mukherjee 2008; Connor et al. 2005; You et al. 2014) and have been investigated as carriers for various anticancer agents (Cheng et al. 2008; Ghosh et al. 2008; Gibson et al. 2007; Kim et al. 2009; Lee et al. 2009).

Hollow gold nanoparticles (HGNPs) are core-shelled gold nanostructures with a hollow interior (Melancon et al. 2011). Attached to a homing ligand, polyethylene glycol (PEG)-coated HGNPs can be administered systemically for active targeting of tumor cells (Braun et al. 2009; Melancon et al. 2008). The strong SPR absorption at near-infrared (NIR) wavelengths induces potent photothermal effects for photoacoustic tomography (Lu et al. 2010a, 2011) and photothermal ablation applications (Melancon et al. 2008). HGNPs have also been shown to mediate light-triggered release of chemotherapeutic agents (You et al. 2010) and therapeutic small-interfering RNAs (siRNA) (Braun et al. 2009; Lu et al. 2010b).

Several mechanisms are used to trigger the release of drugs from Au-based photothermal-converting nanoparticles. In doxorubicin-loaded HGNPs, the release of doxorubicin is activated by NIR light through thermal desorption of doxorubicin from the Au surface (You et al. 2010). Yavuz et al. (2009) took advantage of the caging effect of thermally responsive polymer-coated Au nanocages to control drug release. NIR light-triggered release of DNA is thought to result from the breakage of Au–S bonds by a thermal energy transfer mechanism due to localized absorption of laser energy by HGNPs to generate thermal or electron heating (Chen et al. 2006; Jain et al. 2006).

**Scheme 1:**
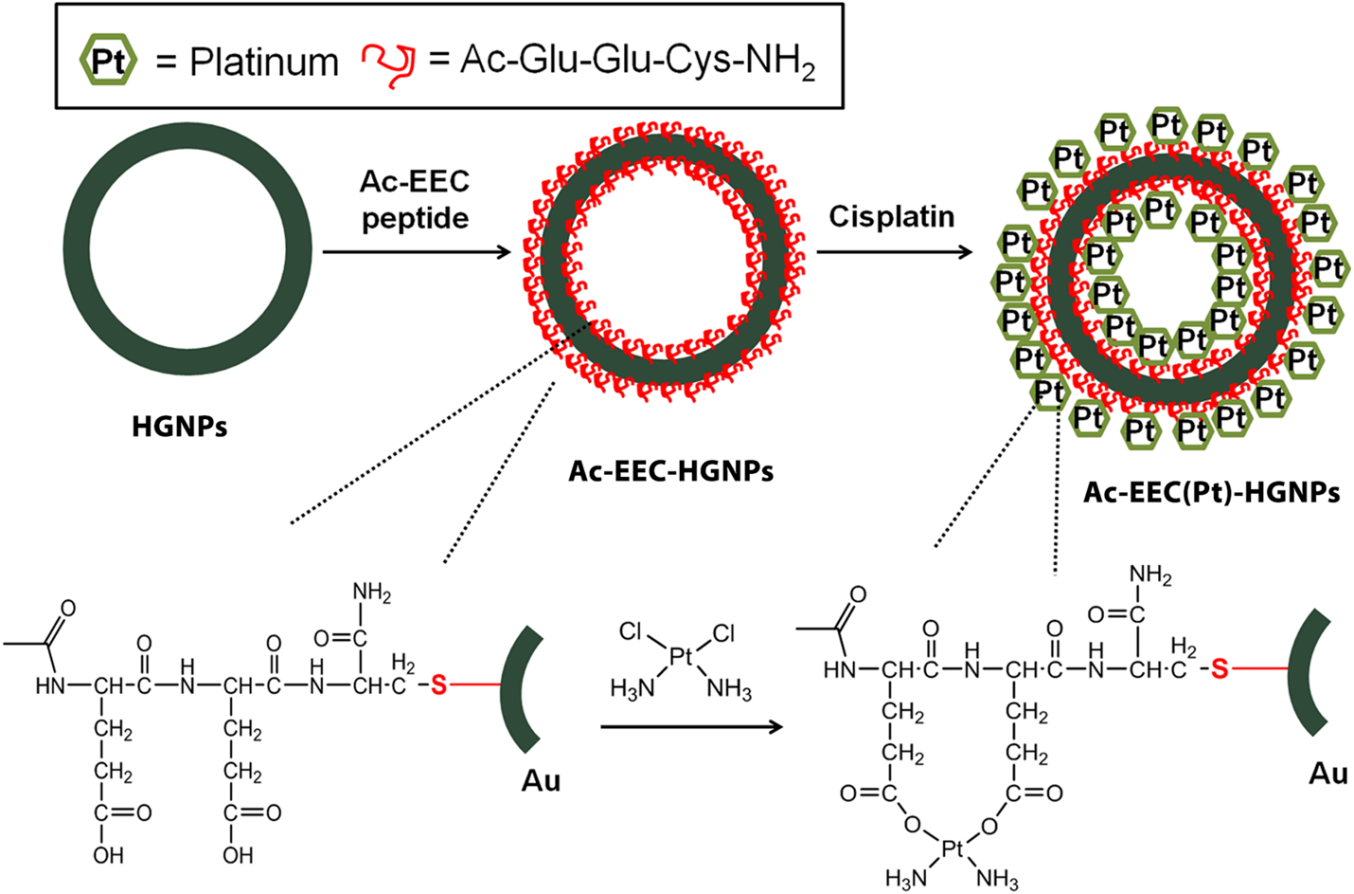
Schematic illustrations of cisplatin-loaded HGNPs. The HGNPs incorporating cisplatin (Pt) are spontaneously formed via a ligand exchange reaction of Pt(II) from the chloride to the carboxylates in the Ac-EEC peptide in distilled water

In this work, we developed a NIR light-triggered drug release system that could release platinum [Pt(II)]-based chemotherapeutic agents through the same mechanism. Cisplatin is one of the most effective and widely used anticancer drugs. Several research groups have reported the formation of Pt complexes that contain Pt–N/Pt–Cl or Pt–N/Pt–O coordination bonds with two Pt–N bonds in the *cis* position (Cheng et al. 2009; Dhar and Lippard 2009; Nishiyama et al. 2003). We designed a tripeptide, acetyl-Glu-Glu-Cys-NH_2_ (Ac-EEC), to serve as both a Pt(II) chelating agent and a linker to HGNPs. The peptide was covalently bound to HGNPs via the cysteine residue to form a gold-thiol (Au–S) bond. Pt(II)-incorporated HGNPs were prepared through complexation of cisplatin and Ac-EEC, where a coordination bond forms between the Pt(II) ion and carboxylate groups in the side chains of glutamic acid residues (Scheme 1). It is demonstrated that irradiation of folate receptor-targeted, Pt(II)-loaded HGNPs, i.e., F-Ac-EEC(Pt)-HGNPs, with a nanosecond (ns) pulsed laser in the NIR window triggers a rapid release of Ac-EEC(Pt) and enhances the cell mortality. Ac-EEC(Pt) was released from Au surface through oxidation of Cys to a weak Au-binding species cysteic acid (Cya), a mechanism not previously appreciated for laser triggered drug release from Au nanoparticles.

## Methods

### Synthesis of Ac-EEC peptide

The Ac-EEC peptides were synthesized by standard solid-phase methodology using *N*^9^-fluorenyl-methyloxycarbonyl chemistry (Fmoc)-amino acids with acid-labile side chain protecting groups. Solid phase syntheses were carried out on a Prelude automatic peptide synthesizer (Protein Technologies, Inc., Tucson, AZ) using commercially available Rink resin. The resin (0.05–0.1 g) was swollen and washed 5 times with 1.5 mL of dimethylformamide/dichloromethane (DMF)/CH_2_Cl_2_. Fmoc groups were removed by 3 treatments with 1.5 mL of 20% piperidine/DMF for 5 min each. For coupling, 3-fold excesses of Fmoc-amino acids, diisopropylcarbodiimide, and 1-hydroxybenzotriazole were added to 3 mL of DMF/CH_2_Cl_2_. This procedure was repeated once. After coupling and deprotection steps, the resins were washed 3 times with 3 mL of DMF/CH_2_Cl_2_. On completion of the peptide chain elongation, the N-terminal was capped by acetic anhydride, and resins were again washed 3 times with 3 mL of CH_2_Cl_2_ and were treated with a mixture of trifluoroacetic acid:triisopropylsilane:H_2_O (95:2.5:2.5) for 15 min. The combined filtrates were left at room temperature for 1–2 h, and the volumes were reduced in vacuum. Peptides were precipitated in ice-cold ethyl ether, collected by centrifugation, washed 2 times with ethyl ether. The peptides were purified by reverse-phase high-performance liquid chromatography on an Agilent 1200 system (C-18, Vydac, 10 × 250 mm, 10 µm; Agilent Technologies, Santa Clara, CA). Liquid chromatography–high-resolution electrospray ionization mass spectrometry (LC–MS) acquired in the positive ion mode was used to identify the peptides.

### Synthesis and characterization of PEG-Ac-EEC(Pt)-HGNPs and F-Ac-EEC(Pt)-HGNPs

HGNPs were synthesized according to our previously reported procedure (Melancon et al. 2008). Briefly, cobalt nanoparticles were first synthesized by a chemical reduction method in the presence of sodium borohydride. The resulting cobalt nanoparticles served as a sacrificial template for the reduction of chloroauric acid and subsequent deposition of metallic gold on their surfaces. The obtained HGNPs nanoparticles were stabilized with sulfhydryl methoxy polyethylene glycol (MeO-PEG-SH, 5 kDa). Ac-EEC peptide (4 mg/mL, 125 µL) was added to PEG-coated HGNPs (1 mg) in 1 mL of water overnight at room temperature. The mixture was centrifuged and washed with deionized water three times to remove free peptide; the resulting Ac-EEC-HGNPs were mixed with 1 mL of aqueous cisplatin (2.5 mg/mL) and the mixture subjected to shaking at 60 °C in a water-bath for 2 h. The solution was further shaken for 72 h at room temperature and then centrifuged at 7000 rpm for 15 min. The product, Ac-EEC(Pt)-HGNPs, was washed 3 times with deionized water and collected for further characterization.

Folic acid was introduced to the gold surface of Ac-EEC-HGNPs through a thioctic acid–terminated PEG linker (F-PEG-TA) according to previously reported procedures (Lu et al. 2010a). Briefly, Ac-EEC-HGNPs (1 mg) was mixed with both PEG thiol (PEG-SH; 200 µg) and F-PEG-TA (50 µg) in 1 mL water at room temperature overnight to afford the folic acid–conjugated product F-Ac-EEC-HGNPs. Similarly, the non-targeting counterpart PEG-Ac-EEC-HGNPs was synthesized by mixing 250 µg of PEG-SH with Ac-EEC-HGNPs without F-PEG-TA. The resulting conjugates, F-Ac-EEC-HGNPs and PEG-Ac-EEC-HGNPs, were finally complexed with cisplatin to yield F-Ac-EEC(Pt)-HGNPs and PEG-Ac-EEC(Pt)-HGNPs by following the same procedures used for the synthesis of Ac-EEC(Pt)-HGNPs.

Drug payload (Pt to Au ratio) was calculated by determining the amounts of Au and Pt following dissolution of the samples in aqua regia (Cheng et al. 2009). Au and Pt contents were quantified by inductively coupled plasma mass spectroscopy (ICP-MS; Galbraith Laboratories, Inc., Knoxville, TN) on a Varian 810 model (Varian, Inc., Walnut Creek, CA). HNO_3_ (2% v/v) was recorded as a blank solution prior to analysis of each individual sample to monitor memory effects.

High-resolution transmission electron microscopy (HRTEM) micrographs were obtained using JEOL 2100 field emission TEM gun operating at 200 kV (JEOL, Tokyo, Japan). TEM specimens were made by evaporating one drop of sample solution onto ultrathin carbon type-A 200 mesh copper grids (Ted Pella Inc., Redding, CA). Measurements of dynamic light scattering (DLS) were conducted with a Zetaplus (Brookhaven, Inc, Holtsville, NY) in low volume disposable cuvettes and the mean of at least three measurements was taken. The energy-dispersive X-ray spectroscopy (EDS) mapping of gold and platinum from HRTEM was obtained by using a Gatan Imaging Filter (Gatan, Inc., Pleasanton, CA). X-ray photoelectron spectroscopy (XPS) measurements were collected by using a PHI Quantera SXM XPS/ESCA system (ULVAC-PHI, Inc., Kanagawa, Japan). A monochromatic Al X-ray source at 100 W was used with an analytical spot size of 0.15 mm × 1.4 mm and a 45° takeoff angle, with pass energy of 26.00 eV. Unless noted, samples were referenced against an internal Au 4f_7/2_ line at 84.0 eV or Pt 4f_7/2_ line at 71.2 eV. Powder XPS photoelectron lines were referenced against the C 1 s signal at 284.50 eV.

### Release of Pt from PEG-Ac-EEC(Pt)-HGNPs

PEG-Ac-EEC(Pt)-HGNPs (0.4 mg/mL) were dispersed in 2.0 mL deionized water or saline solution in a 5-mL test tube at room temperature. An NIR laser with a peak centered at 808 nm was generated from a master oscillator power amplifier Ti^3+^:sapphire tunable laser (LT-2211A, LOTIS TII, Minsk, Republic of Belarus) pumped by a pulsed Q-switched Nd:YAG laser system (LS-2137/2, LOTIS TII) with the following parameters: wavelength, 532 nm; pulse duration, 15 ns; pulse energy, 400 mJ; laser beam diameter, 7.0 mm. The laser beam was expanded through a concave lens to a spot size of about 1 cm in diameter. A PM50-10 analog optical power meter with an S212A sensor (Thorlabs, Newton, NJ) was used to measure the power of the laser beam at the sample position. At predetermined time intervals, the samples were irradiated with the NIR laser. The HGNPs solutions were subjected to centrifugation (14,000 rpm, 20 min) and the supernatants withdrawn for analysis of Pt before and after NIR laser irradiation by ICP-MS.

In a separate experiment, PEG-Ac-EEC(Pt)-HGNPs were dispersed in 2.0 mL deionized water or saline solution without laser irradiation at room temperature. The Pt concentration was analyzed at predetermined time intervals.

### Identification of Pt-containing compounds released from PEG-Ac-EEC(Pt)-HGNPs

To identify the Pt derivatives released after laser irradiation, Ac-Glu-Glu-Cysteic acid-NH_2_ (Ac-EECya) was synthesized using Rink amide resin and Fmoc chemistry as described above. The complex of cisplatin and Ac-EECya peptide, Ac-EECya(Pt), was synthesized by mixing cisplatin and Ac-EECya in water at 37 °C for 72 h. LC–MS analysis was applied to aliquots collected from both PEG-Ac-EEC(Pt)-HGNPs and F-Ac-EEC(Pt)-HGNPs after laser treatment, and the resulting chromatograms and mass spectra were compared with that of the authentic Ac-EECya(Pt) to confirm the structure of the released Pt-containing species.

### Cytotoxicity

Folate receptor-expressing KB cells were seeded in 96-well plates (2.0 × 10^4^ cells/well) and incubated for 24 h to allow the cells to attach. The cells were exposed to free cisplatin, PEG-Ac-EEC(Pt)-HGNPs, or F-Ac-EEC(Pt)-HGNPs with various Pt concentrations. After 4 h, the cells were irradiated with 15-ns pulsed NIR laser at an output power of 50 mW/cm^2^ (1 min/treatment, 3 treatments in 2 h). The cells were then incubated at 37 °C for an additional 24 or 48 h without washing steps. Cell survival efficiency was measured by the (3-[4,5-dimethylthiazol-2-yl]-2,5 diphenyl tetrazolium bromide), or MTT, assay (Sigma, St Louis, MO) according to manufacturer-suggested procedures. The data are reported as the means of triplicate measurements.

In a separate experiment, KB cells were seeded onto a 96-well plate at a density of 20,000 cells/well 24 h before the experiment. Cells were washed 3 times with Dulbecco modified essential medium (DMEM) without phenol red. Cells were incubated with 100 µL of DMEM containing F-Ac-EEC-HGNPs (17 µM of Au) or F-Ac-EEC(Pt)-HGNPs (17 µM of Au and 5 µM of Pt) at 37 °C for 4 h. The cells were then washed 3 times with phosphate-buffered saline solution to remove unbound nanoparticles. After cells were resupplied with DMEM containing 10% fetal bovine serum, they were irradiated with 15-ns pulsed NIR laser light centered at 808 nm at an output power of 50 mW/cm^2^ (1 min/treatment, 3 treatments in 2 h) and then incubated at 37 °C. Twenty after laser treatment, cells were washed 3 times with Hanks balanced salt solution and stained with calcein AM (Life Technologies, Grand Island, NY) for visualization of live cells. Untreated cells were used as controls. Cells were examined on a Zeiss Axio Observer. Z1 fluorescence microscope (Carl Zeiss MicroImaging GmbH, Göttingen, Germany) equipped with a filter set specific for excitation/emission wavelengths at 494/517 nm for calcein staining.

## Results and discussion

### Characterization of PEG-Ac-EEC(Pt)-HGNPs

**Fig. 1:**
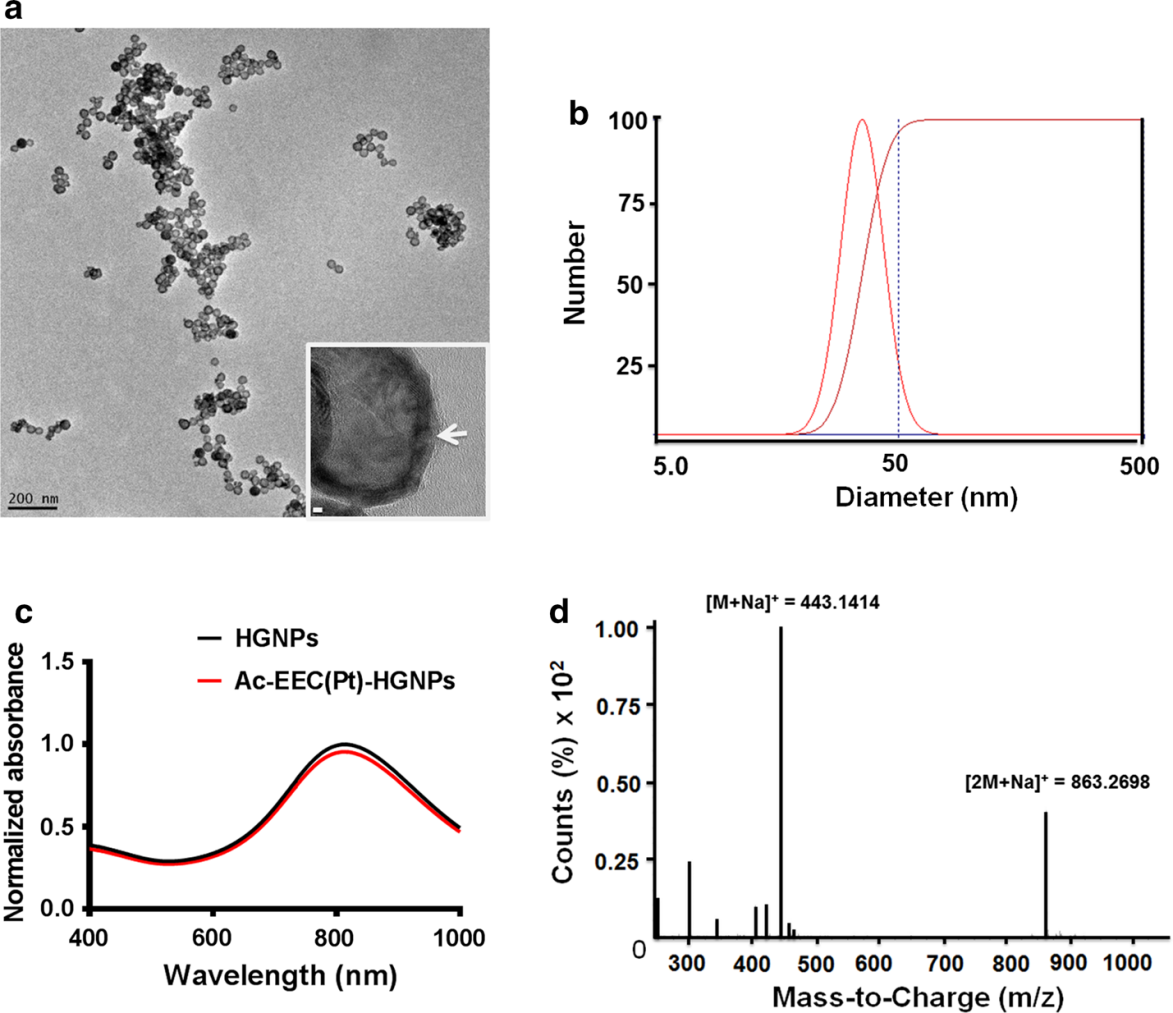
Characterization of HGNPs loaded with Pt. **a** Representative HRTEM micrograph of Ac-EEC(Pt)-HGNPs. Arrow, nanopore on Au shell. Scale bar in inset, 2 nm. **b** Representative DLS curve showing size distribution of Ac-EEC(Pt)-HGNPs. **c** UV–Vis-NIR spectrum of HGNPs and Ac-EEC(Pt)-HGNPs. **d** Mass spectrum of Ac-EEC peptide with positive ion mode

**Fig. 2:**
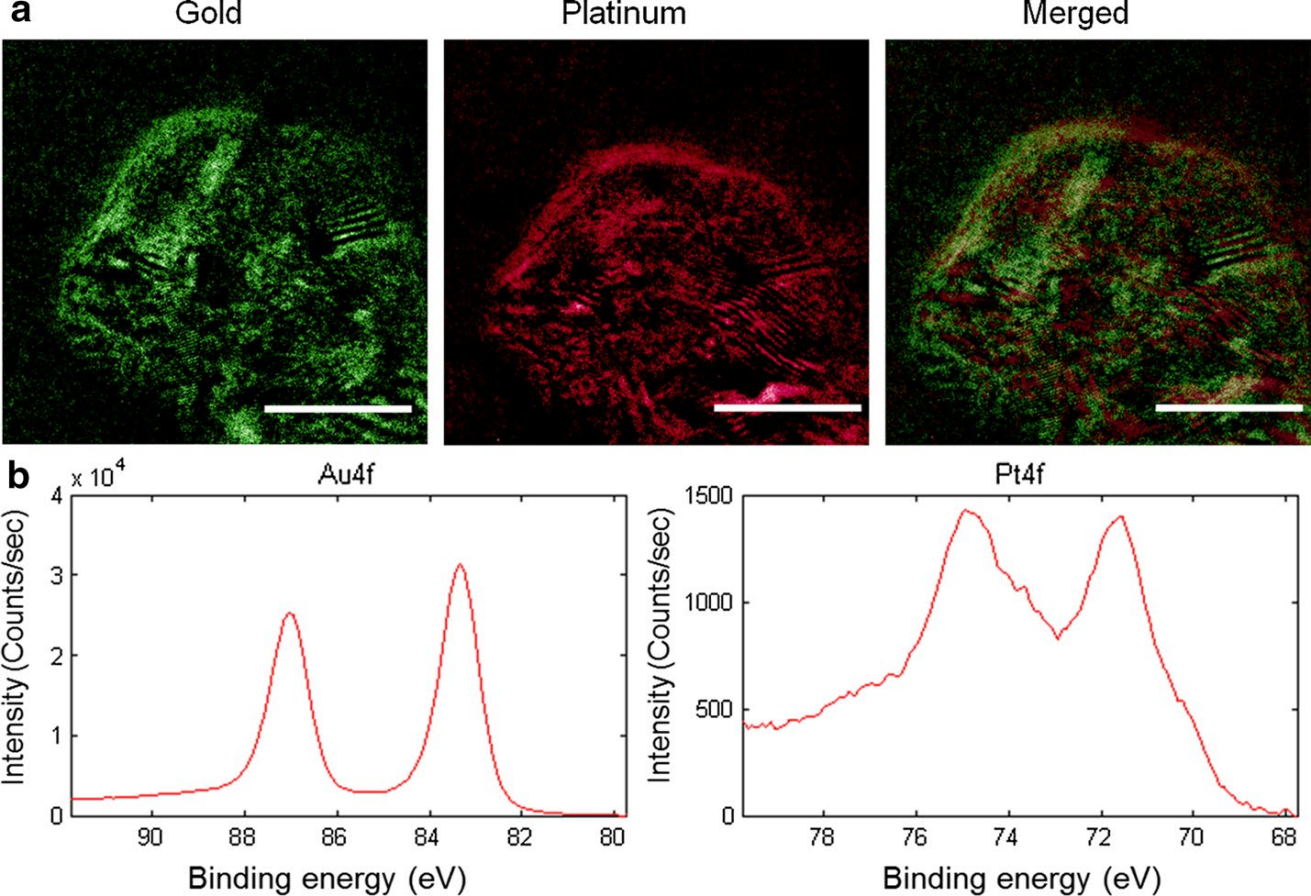
Characterization of HGNPs loaded with Pt. **a** HRTEM/EDS micrographs mapping distribution of Au and Pt elements in Ac-EEC(Pt)-HGNPs. **b** XPS analysis of Au and Pt in Ac-EEC(Pt)-HGNPs

In PEG-Ac-EEC(Pt)-HGNPs, Pt loading was achieved through a linker peptide, Ac-EEC, which contained 2 Glu units for Pt complexation and a Cys residue for conjugation to HGNPs. HRTEM illustrates that the typical Ac-EEC(Pt)-HGNPs had a diameter of ~ 43.9 ± 1.2 nm (n = 200), with a hollow core and a shell of ~ 3–4 nm in thickness, and was porous on the surface (Fig. 1a). The size distribution of PEG-coated HGNPs was determined by dynamic light scattering; the hydrodynamic diameters of PEG-coated HGNPs varied from 20 to 65 nm peaking at ~ 45 nm (Fig. 1b). The extinction spectra showed the plasmon resonance peak at 808 nm for both plain HGNPs and drug-loaded Ac-EEC(Pt)-HGNPs (Fig. 1c). The LC–MS spectrum shows that the synthetic Ac-EEC tripeptide had an exact mass of 420.1315, giving rise to mass-to-charge (m/z) values of 443.1414 for [M + Na]^+^ and 863.2698 for [2M + Na]^+^ (Fig. 1d). The presence of pores on the Au shell allowed Ac-EEC peptide and Pt(II) ions to diffuse into the interior of the HGNPs, as was the case for doxorubicin (You et al. 2010). The notion that Pt(II) bound to both the inner and the outer surfaces of the shell layer was supported by the observed distribution of Pt throughout HGNPs, as shown on EDS analysis (Fig. 2a). The presence of both Pt(II) and Au in HGNPs was confirmed by XPS, which showed the presence of both Pt4f and Au4f peaks (Fig. 2b). Because Pt(II) was bound to both inner and outer surfaces of HGNPs, the resulting HGNPs-Pt had a relatively high Pt(II) payload. PEG-Ac-EEC(Pt)-HGNPs contained 21.9% by weight of Pt(II) as revealed by ICP-MS analysis.

### NIR laser-triggered Pt(II) release

**Fig. 3:**
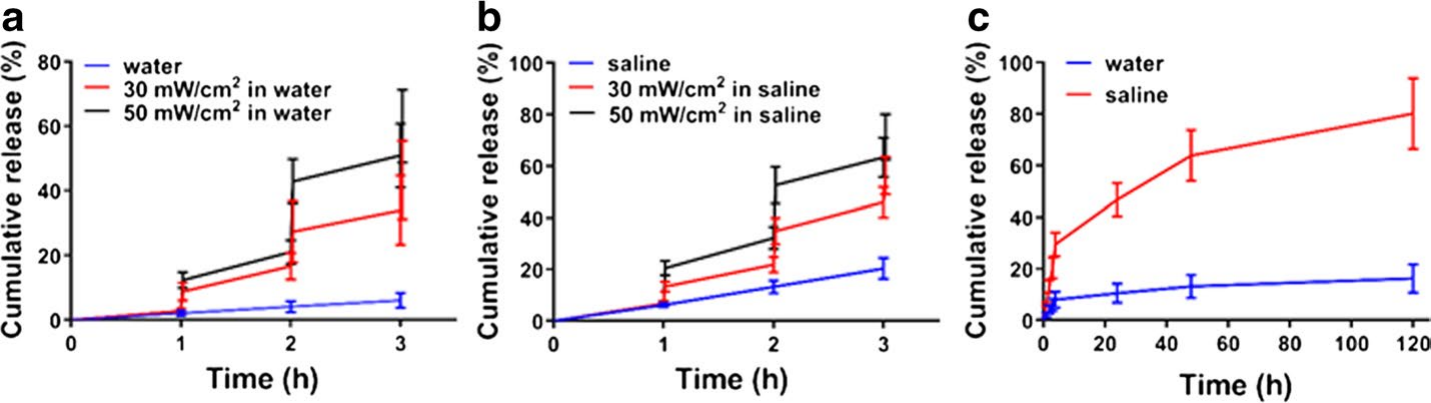
Short pulsed laser induced rapid release of Pt. **a** Pt(II) release from Ac-EEC(Pt)-HGNPs in deionized water with or without NIR laser treatment at 30 or 50 mW/cm^2^. **b** Pt(II) release from Ac-EEC(Pt)-HGNPs in saline solution with or without NIR laser treatment at 30 or 50 mW/cm^2^. In the laser treatment groups, Nd:YAG pulsed laser (at 808 nm) was applied at the time of the arrows indicated for 1 min duration. **c** Sustained release of Pt in water or saline solution without laser irradiation. Data are presented as means and standard deviation of 3 experiments

We next investigated Pt(II) release from PEG-Ac-EEC(Pt)-HGNPs with or without laser irradiation in the presence or absence of chloride ions. Figure 3 illustrates that Pt-containing compounds were released from PEG-Ac-EEC(Pt)-HGNPs upon NIR laser irradiation. In deionized water, the cumulative release, defined as the percentage of released Pt, increased from 2.8 to 8.8% during the first 1-min NIR laser exposure at an output power of 30 mW/cm^2^. Release of Pt slowed drastically when the NIR laser was switched off over the next 1 h of incubation. Similar results were observed when the laser treatment protocol was repeated beginning at 2 h. The cumulative release of Pt increased from 16.7 to 27.4% during the second 1-min treatment cycle. During the third treatment cycle (beginning at 3 h), the cumulative release of Pt increased from 34.0 to 43.3%. These data suggested that rapid Pt(II) release from PEG-Ac-EEC(Pt)-HGNPs could be triggered by pulsed NIR laser.

The amount of Pt(II) released from PEG-Ac-EEC(Pt)-HGNPs could be modulated by controlling the laser power. Thus, increasing laser power from 30 to 50 mW/cm^2^ (but keeping the treatment duration to 1 min) resulted in an increase of the cumulative Pt(II) release from 8.8 to 12.4% during the first treatment cycle, from 27.4 to 42.9% during the second treatment cycle, and from 43.3 to 60.0% during the third treatment cycle (Fig. 3a). Without laser treatment, the cumulative Pt(II) release was only 6.1% during the first 3 h. Similar laser-triggered Pt(II) release profiles were observed when PEG-Ac-EEC(Pt)-HGNPs was incubated in saline solution (Fig. 3b). However, the presence of chloride ions increased the release of Pt from the 6.1% observed in water to 20.4% over the 3 h incubation period. In saline solution, the total amounts of Pt(II) released following 3 cycles of NIR laser treatment (1 min treatment for each cycle) at 30 and 50 mW/cm^2^ output powers were 56.3 and 71.3%, respectively.

To further evaluate the effects of ion exchange on the Pt(II) release profiles, the release of Pt from PEG-Ac-EEC(Pt)-HGNPs without NIR laser exposure was recorded for 120 h. About 29.6% of Pt was released from PEG-Ac-EEC(Pt)-HGNPs within the first 4 h of incubation in saline solution. This was followed by a slower release rate, with another approximately 50% of the Pt(II) released over the next 5 days, resulting in a cumulative Pt(II) release of 80.2% after 120 h (Fig. 3c). In contrast, the total amount of Pt(II) released in deionized water was only 16.3% over the same time period. In PEG-Ac-EEC(Pt)-HGNPs, a coordination bond formed between the Pt(II) ion and carboxylic groups in the side chains of glutamic acid residues. This design was also used in Pt-containing polymeric micelles that were prepared through the complexation between cisplatin and PEG-poly(glutamic acid) block copolymers (Nishiyama et al. 2003). The Pt-containing micelles showed slow dissociation into unimers, accompanied with a sustained cisplatin release in physiological saline solution due to the inverse ligand exchange reaction of Pt(II) from the carboxylates in the copolymer to the chloride ions. However, very little Pt was released from the micelles in distilled water (Nishiyama et al. 2003). Our findings agree with the results from Pt-containing polymeric micelles that showed sustained Pt(II) release profiles in the presence of chloride ions.

### Modes of Pt(II) release upon laser irradiation

**Fig. 4:**
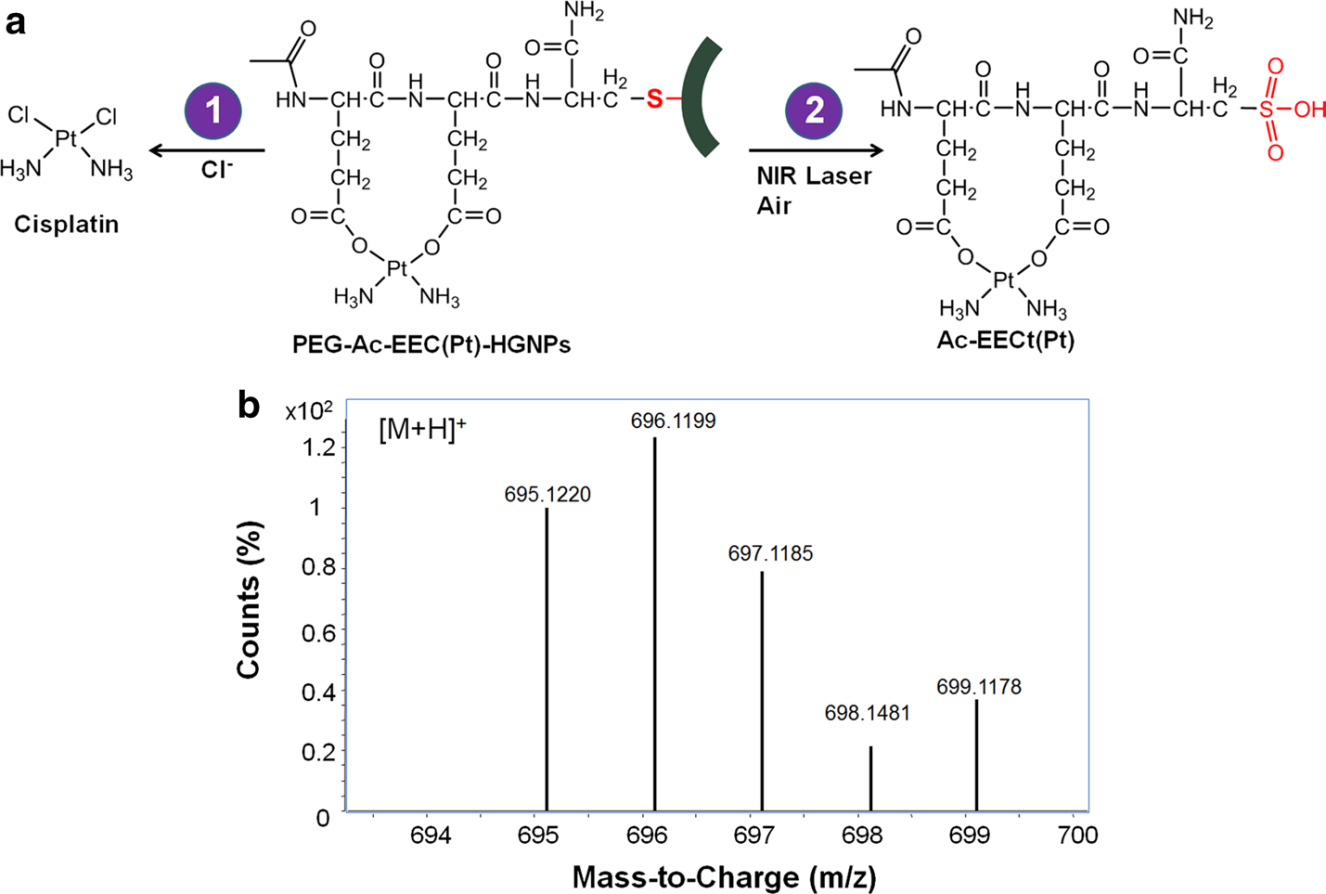
Proposed modes of Pt(II) release from Ac-EEC(Pt)-HGNPs. **a** Schematic illustrations of Pt(II) release modes. In release mode 1, Pt(II) complexed to Ac-EEC attached to HGNPs dissociates, accompanied by sustained release of Pt via an inverse ligand exchange reaction from Glu carboxylates to the chloride ions in physiological saline solution. In release mode 2, rapid release of Pt-complexed to Ac-EEC is triggered by NIR laser irradiation through cleavage of Au–S bonds. The compound Ac-EECya(Pt) detaches from the Au surface as the Cys is oxidized to cysteic acid (Cya). **b** Mass spectrum of Ac-EECya-Pt(II) released from HGNPs upon NIR laser irradiation. LC–MS analysis of the released Ac-EECya-Pt was done with ESI positive ion mode. The isotopic [M + H]^+^ peaks and the corresponding abundance match those of calculated values for Ac-EECya-Pt

The results just described demonstrate two Pt(II) release mechanisms from PEG-Ac-EEC(Pt)-HGNPs. First, Pt could be released from PEG-Ac-EEC(Pt)-HGNPs without NIR laser treatment in the presence of chloride ions or other anionic species that compete with carboxylates for Pt complexation, resulting in sustained release of Pt species. Second, Pt(II) release could be induced rapidly from PEG-Ac-EEC(Pt)-HGNPs upon NIR laser irradiation (Fig. 4a).

Direct Au–S bond cleavage has been thought to underlie fast pulsed laser-triggered release of thiolated plasmid DNA for gene transfection and siRNA for RNA interference (Braun et al. 2009; Chen et al. 2006; Lu et al. 2010a; Takahashi et al. 2005; Wijaya et al. 2009). Similarly, the Ac-EEC(Pt) peptide complexes may be cleaved from HGNPs owing to a photothermal effect of HGNPs that confines thermal energy to the immediate vicinity of HGNPs with short pulsed laser. Under such conditions, Au–S bonds could be cleaved with either thermal energy transfer (Chen et al. 2006) or confined hot electrons (Jain et al. 2006). In the latter case, the hot electrons from the Au plasmons destabilizes Au–S bonds before the electrons had thermalized with the lattice of the material (Jain et al. 2006). Alternatively, Au–S bonds may break because of oxidative desorption in which sulfhydryl in Cys is converted to sulfonate in Cya that has substantially weaker bonding with Au than the corresponding thiolate (Huang et al. 1994) (Fig. 4a). To account for this possibility, aliquots from PEG-Ac-EEC(Pt)-HGNPs after exposure to ns-pulsed laser in air was subjected to LC–MS analysis. LC–MS spectra showed prominent peaks with m/z values ranging from 695.1220 to 699.1178 (Fig. 4b). These isotopic peaks matched calculated values for the [M + H]^+^ peaks of Ac-EECya(Pt), confirming that oxidization of the Au–S bond to Au–S(=O)(=O) could be an important mechanism underlying Pt(II) release from HGNPs with ns-pulsed laser.

### In vitro anticancer effects

**Fig. 5:**
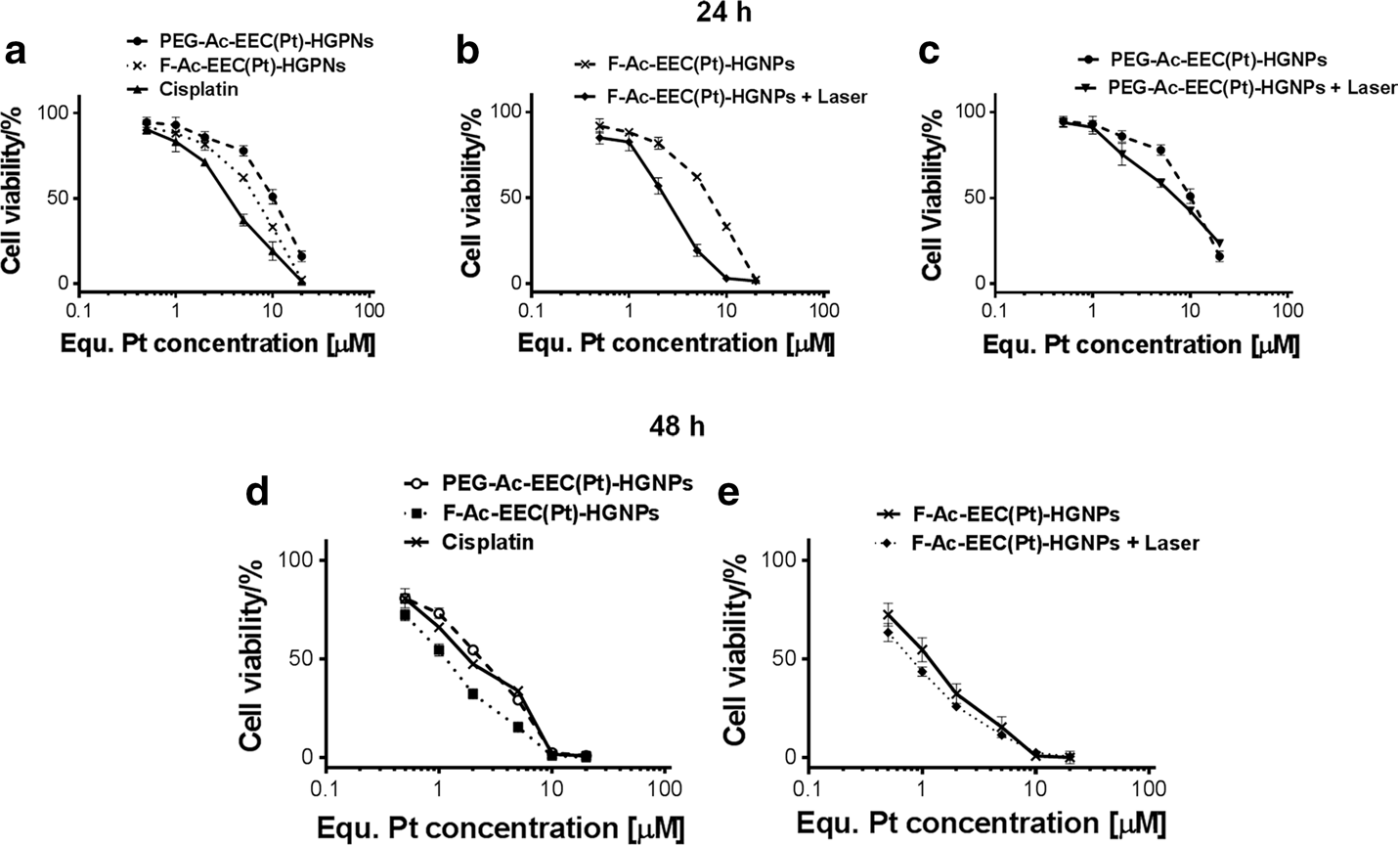
Treatment with F-Ac-EEC(Pt)-HGNPs plus NIR laser exposure has greater cell mortality than F-Ac-EEC(Pt)-HGNPs alone after 24 h incubation. Viability of folate receptor–overexpressing KB cells was determined by MTT assay after treatments shown after 24 h (**a**–**c**) and 48 h (**d**, **e**) of incubation

We used folate receptor-overexpressing KB cells to investigate cellular targeting of folic acid–conjugated Ac-EEC(Pt)-HGNPs to tumor cells (Werner et al. 2011). After 24 h of incubation, F-Ac-EEC(Pt)-HGNPs was more cytotoxic against KB cells than non-targeted PEG-Ac-EEC(Pt)-HGNPs. However, F-Ac-EEC(Pt)-HGNPs had lower cytotoxicity than the equivalent dose of free cisplatin. The calculated IC_50_ values for free cisplatin, F-Ac-EEC(Pt)-HGNPs, and PEG-Ac-EEC(Pt)-HGNPs were 3.37 µM (95% confidence interval [CI] 2.67–4.26 µM), 5.68 µM (95% CI 4.42–7.31 µM), and 9.73 µM (95% CI 7.54–12.56 µM), respectively (Fig. 5a). Laser treatment had a greater effect on cells treated with F-Ac-EEC(Pt)-HGNPs than on cells treated with non-targeted PEG-Ac-EEC(Pt)-HGNPs (Fig. 5b, c). This is probably because the nanoparticles internalized into the cells via endocytosis were first sequestered in the endosomal compartments instead of the cytoplasm, which limited the transport of the Pt agent to cell nuclei to form DNA adducts. In addition, the rate of Pt(II) release from HGNPs was relatively slow without laser exposure. In contrast, free cisplatin can passively diffuse through the cell membrane into the cytoplasm and quickly accumulate in the cell nuclei (Cheng et al. 2009).

After 48 h of incubation, PEG-Ac-EEC(Pt)-HGNPs and F-Ac-EEC(Pt)-HGNPs exhibited IC_50_ values of 2.02 µM (95% CI 1.66–2.45 µM) and 1.06 µM (95% CI 0.91–1.23 µM), respectively (Fig. 5d), lower than the IC_50_ values of the corresponding compounds after 24 h of incubation (9.73 µM for PEG-Ac-EEC(Pt)-HGNPs, 5.68 µM for F-Ac-EEC(Pt)-HGNPs). These values were similar to the IC_50_ value of cisplatin (1.8 µM) after 48 h of incubation, suggesting that the benefit of targeted delivery diminished over time. Similar cytotoxicity was also observed after treatment with F-Ac-EEC(Pt)-HGNPs alone (IC_50_ = 1.06 µM) and F-Ac-EEC(Pt)-HGNPs plus laser (IC_50_ = 0.75 µM, 95% CI 0.67–0.85 µM) (Fig. 5e) after the 48-h incubation period. These data suggest that the cytotoxic effect of the Pt(II) released from F-Ac-EEC(Pt) HGNPs became the predominant mechanism of increasing cell mortality after a longer incubation period.

**Fig. 6:**
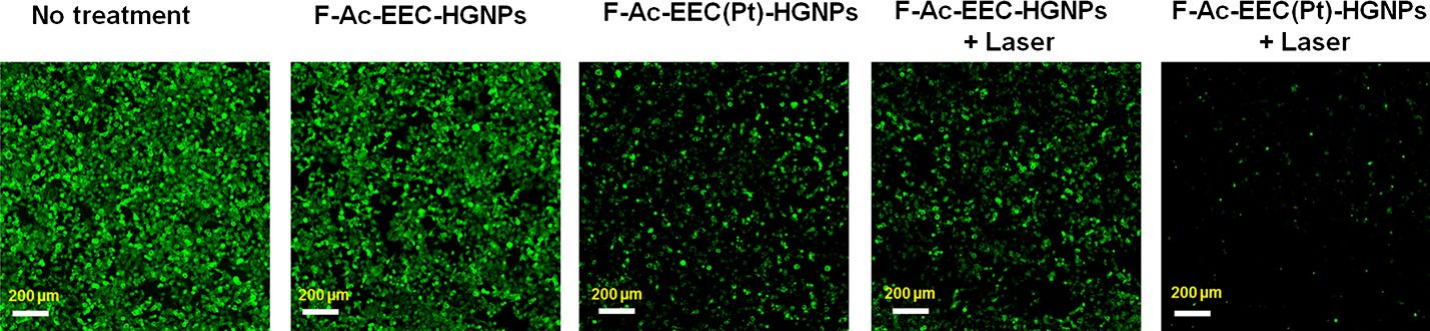
Microphotographs showing enhanced cell killing with combined F-Ac-EEC-HGNPs and laser treatments. KB cells were exposed to F-Ac-EEC-HGNPs without Pt loading or F-Ac-EEC(Pt)-HGNPs with Pt loading for 4 h. The cells were or were not irradiated with a pulsed laser at 808 nm (50 mW/cm^2^; 1 min). After washing steps and an additional 20 h incubation, cells were stained with calcein AM. Green fluorescence represents viable cells. Bar, 200 μm

To further evaluate the role of targeting ligand on Ac-EEC(Pt)-HGNPs in vitro, live KB cells were stained with calcein AM 20 h after laser treatment (Fig. 6). This experiment differed from the previous cytotoxicity study in that cells were only exposed to nanoparticles for 4 h, then nanoparticles were removed by repeated washing steps before laser treatment was instituted. Under such conditions, both F-Ac-EEC-HGNPs and F-Ac-EEC(Pt)-HGNPs mediated greater effect on cell mortality against KB cells when combined with laser treatment than treatment with the corresponding nanoparticles alone (Fig. 6). Cells treated with F-Ac-EEC(Pt)-HGNPs plus laser exhibited ~ 85% decrease in fluorescence intensity compared to untreated control cells. Cells treated with F-Ac-EEC(Pt)-HGNPs alone or F-Ac-EEC-HGNPs (without Pt) plus laser showed moderately lower viability than untreated control cells (~ 40–45% decrease in fluorescence intensity). There was no difference in viability between cells treated with F-Ac-EEC-HGNPs alone and untreated cells, indicating the low cytotoxicity of F-Ac-EEC-HGNPs when these nanoparticles were not loaded with Pt. These results suggest that laser-triggered release of Pt(II) from F-Ac-EEC(Pt)-HGNPs increased the cell mortality of photothermolysis.

## Conclusions

In this study, we synthesized and characterized Pt(II)-loaded HGNPs using a tripeptidelinker, Ac-Glu-Glu-Cys-NH_2_, designed to serve both as a Pt(II) chelating agent and a linker to HGNPs. The resulting Pt-HGNPs nano-carrier system displayed 2 release mechanisms. In the presence of chloride ions, the Pt(II) was released in a sustained fashion by an ion-exchange process. Upon NIR laser irradiation, oxidative desorption occurred that resulted in cleavage of Au–S bonds and rapid release of soluble Pt(II) compounds. F-Ac-EEC(Pt)-HGNPs combined with laser irradiation displayed greater effects on cell mortality than compared with the sole F-Ac-EEC(Pt)-HGNPs treatment over a 24h incubation period.

In the clinic, photothermal ablative therapy using a Nd:YAG laser has been studied as a palliative treatment for inoperable tumors or as an alternative to more radical surgery (Paiva et al. 1998, 2002, 2005). During photothermal ablation, the tumor periphery adjacent to normal tissues can get a suboptimal thermal dose, causing reversible hyperthermic damage that can result in tumor recurrence (Paiva et al. 2005). The dual photothermal-chemotherapy approach exemplified by F-Ac-EEC(Pt)-HGNPs that release therapeutic agents upon laser irradiation may offer greater antitumor efficacy than photothermolysis alone mediated by plain HGNPs. Further studies on the pharmacokinetics, biodistribution, and antitumor activity of the multimodal Pt(II)-loaded HGNPs drug delivery system with NIR laser therapy are warranted.
